# An Efficient Modelling-Simulation-Analysis Workflow to Investigate Stump-Socket Interaction Using Patient-Specific, Three-Dimensional, Continuum-Mechanical, Finite Element Residual Limb Models

**DOI:** 10.3389/fbioe.2018.00126

**Published:** 2018-09-19

**Authors:** Ellankavi Ramasamy, Okan Avci, Beate Dorow, Sook-Yee Chong, Leonardo Gizzi, Günter Steidle, Fritz Schick, Oliver Röhrle

**Affiliations:** ^1^Department of Biomechatronic Systems, Fraunhofer-Institut für Produktionstechnik und Automatisierung (Fraunhofer IPA), Stuttgart, Germany; ^2^Diagnostische und Interventionelle Radiologie, Sektion für Experimentelle Radiologie, Department für Radiologie, Universitätsklinikum Tübingen, Tübingen, Germany; ^3^Institut für Mechanik (Bauwesen), Universität Stuttgart, Stuttgart, Germany; ^4^Stuttgart Centre for Simulation Sciences, Universität Stuttgart, Stuttgart, Germany

**Keywords:** continuum-mechanics, diffusion tensor MRI, skeletal muscle, transfemoral amputation, injury

## Abstract

The lack of an efficient modelling-simulation-analysis workflow for creating and utilising detailed subject-specific computational models is one of the key reasons why simulation-based approaches for analysing socket-stump interaction have not yet been successfully established. Herein, we propose a novel and efficient modelling-simulation-analysis workflow that uses commercial software for generating a detailed subject-specific, three-dimensional finite element model of an entire residual limb from Diffusion Tensor MRI images in <20 min. Moreover, to complete the modelling-simulation-analysis workflow, the generated subject-specific residual limb model is used within an implicit dynamic FE simulation of bipedal stance to predict the potential sites of deep tissue injury. For this purpose, a nonlinear hyperelastic, transversely isotropic skeletal muscle constitutive law containing a deep tissue injury model was implemented in LS-DYNA. To demonstrate the feasibility of the entire modelling-simulation-analysis workflow and the fact that detailed, anatomically realistic, multi-muscle models are superior to state-of-the-art, fused-muscle models, an implicit dynamic FE analysis of 2-h bipedal stance is carried out. By analysing the potential volume of damaged muscle tissue after donning an optimally-fitted and a misfitted socket, i.e., a socket whose volume was isotropically shrunk by 10%, we were able to highlight the differences between the detailed individual- and fused-muscle models. The results of the bipedal stance simulation showed that peak stresses in the fused-muscle model were four times lower when compared to the multi-muscle model. The peak interface stress in the individual-muscle model, at the end of bipedal stance analysis, was 2.63 times lower than that in the deep tissues of the stump. At the end of the bipedal stance analysis using the misfitted socket, the fused-muscle model predicted that 7.65% of the residual limb volume was injured, while the detailed-model predicted 16.03%. The proposed approach is not only limited to modelling residual limbs but also has applications in predicting the impact of plastic surgery, for detailed forward-dynamics simulations of normal musculoskeletal systems.

## 1. Introduction

When designing and fitting prosthetic devices, prosthetists try to cater to the unique needs and desires of a patient. Those prosthetic devices that are perceived as good prosthetic fits provide the ability to walk comfortably, which should be as close as possible to normal gait, i.e., someone with a healthy residual limb (Legro et al., 1999). However, studies indicate that prosthetic fit varies, not only from one prosthetist to another, but also within the same prosthetist (Boone et al., 2012; Kobayashi et al., 2015). This indicates that most fits are sub-optimal, and thereby impede the acceptance of prosthetic devices, affect gait, and may lead to pain and injury (Klute et al., 2001; Van Velzen et al., 2005). Until 2008, both computational and experimental studies focussed their efforts towards reducing the socket-stump interface pressure, which was considered to be the most critical factor in deciding the comfort of a prosthesis. The contribution of internal tissue strains towards the evolution of deep tissue injuries was first investigated by Gefen et al. (2008) and Portnoy et al. (2008). These tissue injuries originate deep within the limb and propagate towards the outer skin, making their timely prognosis difficult and dangerous. On the other hand, there exist continuum-mechanical approaches and finite element (FE) simulations that have been extensively used to predict the response of various biological tissues to external stimuli without invasive experiments (e.g., Röhrle et al., 2012; Miga, 2016; Budday et al., 2017; Heidlauf et al., 2017). This makes FE analyses (FEA) a suitable tool to understand internal soft tissue strains in residual limbs. However, to succeed in using FE simulations for investigating and ultimately designing prosthetic devices, if deep tissue injuries can be strongly reduced or eliminated, one requires an efficient tool to construct patient-specific limb models, e.g., from MRI or CT scans and appropriate loading conditions such as the ones that occur during bipedal stance or locomotion.

Over the last two decades, medical scans have been used to generate patient-specific FE models (cf. Zachariah et al., 1996; Portnoy et al., 2008; Zhang et al., 2013; Sengeh et al., 2016; Cagle et al., 2018). In these models, the soft tissues were segmented at most into one or two layers, i.e., muscle-fat complex (Portnoy et al., 2008; Cagle et al., 2018), and fused-muscle and skin (Sengeh et al., 2016), which we refer to in the following as fused-muscle models. The justification provided for such a segmentation was that the small volume of the residuum comprised mostly of a muscle lump. The experimental-numerical validations of the proposed geometry were performed using interface pressure maps by Portnoy et al. (2008) and with indenters by Sengeh et al. (2016). However, it remains to be seen if such minimally-segmented models can also be validated in case of transfemoral residua, where the volume of soft tissues is high, and segmenting the stump into a two-layered model might lead to problems in fitting the material parameters. The necessity of highly detailed human models based on medical-imaging data have been emphasised by several authors. Scheys et al. (2006) and Blemker et al. (2007) emphasise that image-based personalised biomechanical analyses are required to truly understand human movement, motion related disorders, or postoperative gait anomalies, and Fernandez and Pandy (2006) place importance on realistic subject-specific, three-dimensional, individual-muscle FE models. In spite of the emphases on the necessity of detailed muscle models, the review by Dickinson et al. (2017) showed that the proposed FE models were either generic or one- or two-layered segmentation of the limb from MRI and/or CT. Further, they ignore the muscle's anisotropic behaviour. These models were primarily used to study the effects of different loading conditions on the stresses at the socket-residual limb interface. This is despite the fact that recent studies involving patient-specific FE analyses are raising the importance of tissue anisotropy (Sengeh et al., 2016; Dickinson et al., 2017). Such information is, however, not considered to date although it can be obtained, e.g., by utilising diffusion tensor imaging (DT-MRI).

DT-MRI, which is widely used to study the fibrous white matter in the brain (cf. Alexander et al., 2007), can also be used to extract skeletal muscle fibres through a technique called tractography, and the muscle structure thereof (cf. Froeling et al., 2014; Oudeman et al., 2016). The fibre architecture is particularly important for skeletal muscles, as they strongly influence their mechanical behaviour. Without key mechanical properties such as the fibre direction, it would be difficult to study gait and the evolution of internal tissue stresses during locomotion with a prosthesis (cf. Shojaei et al., 2016). In the muscle-driven forward dynamic simulations to study normal and pathological gait (cf. Piazza, 2006), it was stated that knowledge of muscle activations would provide the aetiology of movement disorders, and lead to effective treatments. It therefore follows that patient-specific models that aim to distinguish individual muscles, i.e., resolve them individually, must include muscle fibre orientation if continuum muscle models and the FE method are utilised to perform FE-based biomechanical analyses. Moreover, Dickinson et al. draw in their latest review on the state of utilising FE simulations for analysing the mechanical behaviour of lower limb amputees consensus that computational models used in socket design and tissue viability studies require patient-specific geometry and material models to investigate the internal state of residual limb tissues under load.

To the authors' knowledge, no high-fidelity model of the lower residual limb, in which the muscles are modelled as individual objects exist (the so-called individual-muscle models). More importantly, for the transfemoral case, no conclusive evidence has been provided, which proves that a one- or two-layered segmentation similar to those of the transtibial case is sufficient. Hence, this research proposes a DT-MRI-based modelling-simulation-analysis workflow that requires minimal user intervention, generates highly detailed patient-specific FE models of a residual limb, and uses the detailed model to carry out simulations investigating the difference between individual-muscle and fused-muscle residual limb models. This study is a precursor to future forward dynamics studies involving muscle activations, and mechanical loading of the internal soft tissues during gait.

While this work focuses more on assessing the influence of a particular socket on deep tissue injury (DTI), the same modelling-simulation-analysis workflow can also be utilised for other research questions, e.g., for predicting the result of plastic surgery by simulating, for example, contraction of the planned muscle graft, for detailed forward-dynamics simulations predicting the forces acting within a musculoskeletal system during motion (e.g., as proposed in Röhrle et al., 2017; Valentin et al., 2018), or for analysing neural activity for investigating neural-driven control strategies for prostheses.

## 2. Methods

Given the limitations of existing stump models, the primary objective of this research is to develop a modelling-simulation-analysis workflow to aid in the modelling of a highly accurate three-dimensional, patient-specific, FE residual limb model. The proposed workflow for generating the residual limb model is based on MATLAB to orchestrate the pre- and post-processing of medical image data, to enable data transfer between different software tools, to support the generation of FE mesh of the residuum, and to finally map the muscle fibre information present in the DT-MRI scans onto the generated FE mesh. To complete the modelling-simulation-analysis workflow, the generated, subject-specific residual limb model is used within an implicit dynamic FE simulation of bipedal stance to predict potential regions of the residuum that might be subjected to DTI. This requires appropriate constitutive material models characterising the deformation and injury of soft tissues and reasonable boundary conditions. Here, boundary conditions reflecting bipedal stance are considered. Further, to investigate the necessity of the proposed highly detailed FE model of the residual limb, the dynamic FE simulations of bipedal stance are used to compare the results of the FE analyses between the detailed models and the fused-muscle model of the same residual limb when the same loading conditions are imposed. This is done by comparing the resulting stresses and volumes of regions that are predicted to be affected by DTI.

### 2.1. Data acquisition

The detailed patient-specific residual limb model that is required for the FE analyses shall be derived from a medical image set. For this purpose, DT-MRI scans of the entire residual stump of a left transfemoral amputee were performed at the University Hospital Tübingen, Germany. The MR Examinations were performed on a 3 T MRI whole body scanner (Magnetom Skyra, Siemens Healthcare, Erlangen, Germany), and the volunteer gave written informed consent prior to the examinations. Ethical approval was obtained beforehand (ref: 587/2014BO1).

For signal detection, body array coils of the manufacturer were employed. One coil was positioned below and the other above the thigh stump. A Magnetisation Prepared Rapid Acquisition Gradient-Echo sequence (MP-RAGE) sequence was used to obtain high-resolution 3D data sets of the residual limb, with the socket donned, and without the socket. The sequence parameters were: repetition time (TR) 2,300 ms, echo time (TE) 3.21 ms, inversion time (TI) 974 ms, readout bandwidth 200 Hz/px, flip angle (FA) 8°, in-plane resolution 1.1 × 1.1 mm^2^, matrix size 224 × 448, field of view (FOV) 245 x 491 mm^2^, slice thickness 1.1 mm, 192 sagittal slices and an acquisition time of 4 min and 42 s.

For the DT-MRI scans, a 2D Echo Planar Imaging (EPI) sequence with stimulated echo preparation and fat suppression was applied. The sequence parameters were TR 13,000 ms, TE 36 ms, mixing time (TM) 200 ms, Bandwidth 2,380 Hz/px, resolution 3.2 × 3.2 mm^2^, matrix size 76 × 150, FOV 245 × 491 mm^2^, fractional readout 6/8, slice thickness 3 mm, slice orientation sagittal, 42 slices, 12 diffusion directions, *b*-values 0 and 700 s/mm^2^, 4 acquisitions for each direction and 12 acquisitions for images with *b* = 0 s/mm^2^ (b0 images) and an acquisition time of 13 min and 23 s.

### 2.2. Modelling the residual limb from DT-MRI scans

DT-MRI involves scanning a volume along 6 or more pre-defined gradient directions (cf. Mori and Zhang, 2006). From the diffusion of water molecules in any given voxel of the scanned volume, the variation of the applied magnetic field gradient is measured and its three-dimensional diffusion characteristics determined. These characteristics can be represented by a tensor, the so-called diffusion tensor. In skeletal muscles, this diffusion occurs preferentially along the muscle fibres, thereby revealing the anatomy of the muscles. To obtain a computational subject-specific model suitable for an FE analysis from such data, a workflow was created in MATLAB (R2016a, The Mathworks, Inc., Natick, Massachusetts, United States). The following sections describe the modelling process.

#### 2.2.1. Data preparation

**Figure 1:**
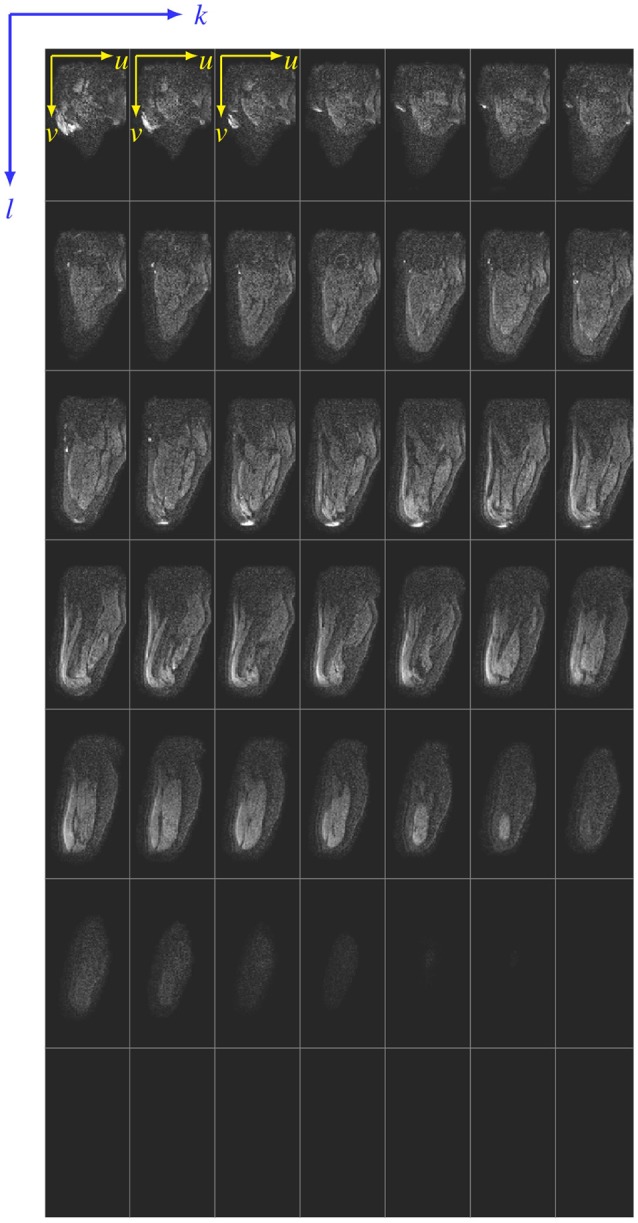
Siemens mosaic file. A sample mosaic image is shown here. For the sake of illustration, the 2D slices that make up the mosaic are separated by thin lines. Any pixel in the mosaic can be accessed using two coordinate systems–the [*k, l*] coordinate that references a 2D slice in the mosaic, and the [*u, v*] coordinate that references a pixel in the 2D slice referenced by [*k, l*].

The DT-MRI scans were obtained in mosaic format (see Figure 1), where each image contained the entire 3D scan volume. For this subject, a total of 60 mosaic images were obtained from 4 acquisitions, where each mosaic image had dimensions of 532 × 1, 050 px, and each 2D slice in the mosaic had dimensions of 76 × 150 px. By first traversing along the columns (*k*-axis) of the mosaic (cf. Figure 1), and stacking each 2D slice one behind another, a 3D representation of the residual limb was created. Each pixel in this mosaic can be accessed by providing two sets of coordinates. The first set of coordinates ([*k, l*]) selects a 2D slice, and the second set of coordinates ([*u, v*]) selects a pixel in this 2D slice. If *i* denotes the intensity of any given pixel in the mosaic, the voxel intensities in the 3D image stack, **S**, is given by

(1) $$\begin{array}{r} \begin{array}{c} \text{S}={\left[i_{uv}\right]}_{kl}=i_{uvm}\\ \text{with }m=N\left(l-1\right)+k, \end{array} \end{array}$$

where *m* can be thought of as the slice number (index) in the constructed 3D volume, *N* is the total number of rows or columns in the mosaic (here, *N* = 7), and indices *k* and *l* index a 2D slice in the mosaic (*k* = *l* = {1, …, 7}). The extents of each 2D slice within the mosaic are 76 × 150 px, i.e., *u* = {1, …, 76} and *v* = {1, …, 150}. In the given example (cf. Figure 1), the last row contains no images (*m* = {1, …, 42}).

**Figure 2:**
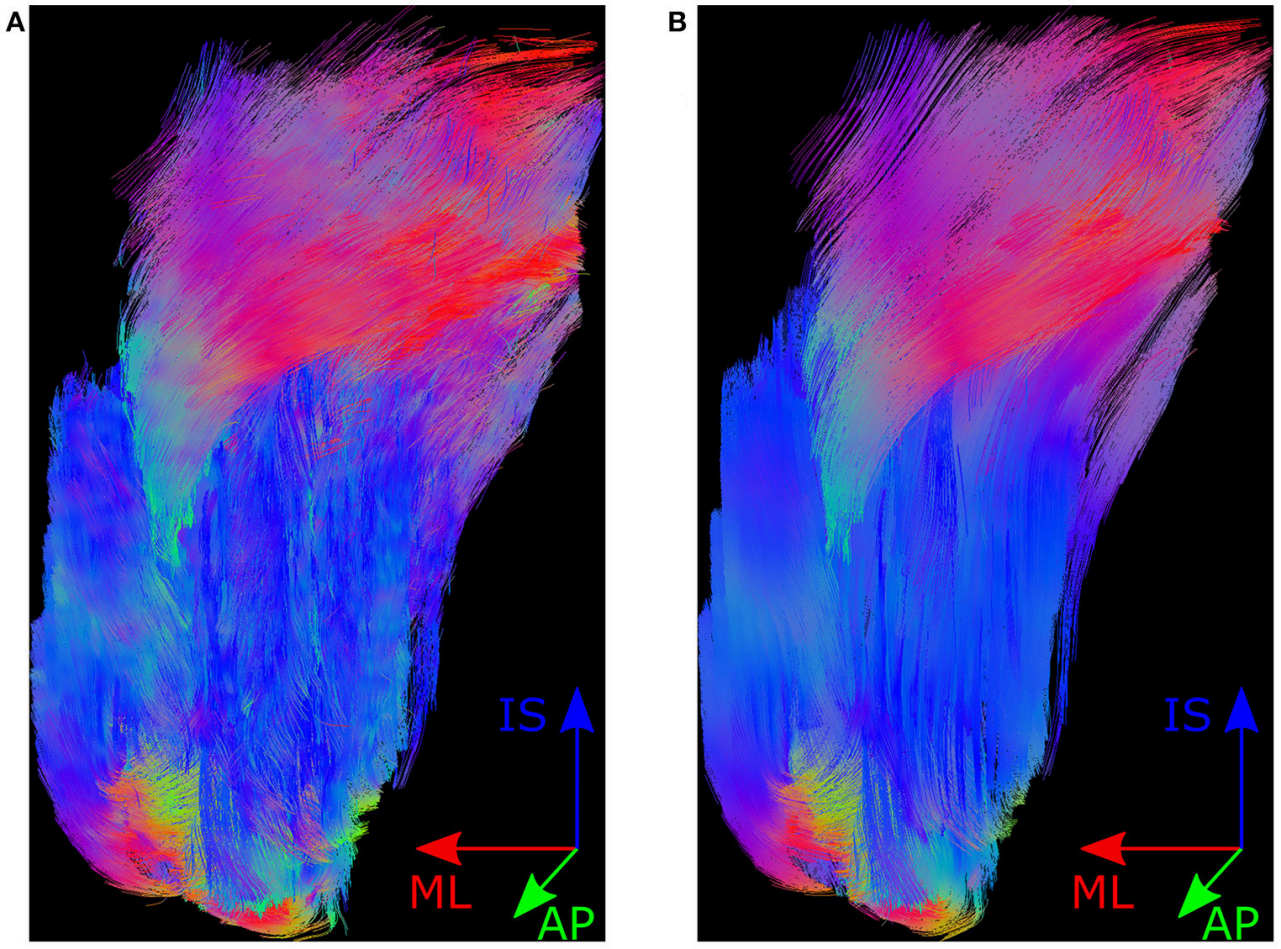
Muscle fibres in MedINRIA. Shown here is the posterior view of the muscle fibre tracts produced by MedINRIA's DTI Track module. Panel **(A)** shows the fibres produced when using the raw NIfTI images as input, and the fibres shown in **(B)** are produced from the de-noised NIfTI images. The colours in fibre tracts indicate the anatomical axis along which they are aligned, i.e., the inferior-superior direction is represented by blue, the medial-lateral axis by red, and the anterior-posterior direction by green.

To reduce noise in the mosaic images, the variance in pixel intensities was reduced by averaging the images over all 4 acquisitions, i.e., the average was taken over 60 images such that we had one image along each gradient direction, and one b0 image, which resulted in a total of 13 mosaics. The “Dicom to nifti converter, nifti tool and viewer” MATLAB toolbox was used to convert these 13 images into a NIfTI file. This NIfTI file was used in MedINRIA's DTI Track module (v1.9.2, 64-bit, ASCLEPIOS Research team, France) to track muscle fibres. The parameters, which were used for fibre tractography are fibre smoothness 10, minimum length of fibres 10, and both FA thresholds were 0. The resulting fibres were wavy due to inherent noise in the scans (cf. Figure 2A). It has been shown that this inherent noise in DT-MRI images is best described by a Rice distribution (cf. Basu et al., 2006). So, a technique using overcomplete local PCA was employed to remove this Rician noise from the NIfTI images (cf. Manjón et al., 2013). The resulting fibres are shown in Figure 2B. DICOM images (DT-MRI-DICOMs) were produced from these averaged and de-noised NIfTI images.

The fibres were segmented into various muscle fibre bundles using MedINRIA's fibre cropping tool. This tool allowed fibres passing through the region of interest, i.e., a cropping box, to be bundled together. Fibres of 11 muscles, which were distinctly visible, were bundled. These were *Adductor Magnus, Biceps Femoris, Gluteus Maximus, Gluteus Minimus, Pectineus, Rectus Femoris, Sartorius, Semimembranosus, Semitendinosus, Vastus Lateralis*, and *Vastus Medialis*. Segmentation of the voxels traversed by each of the above muscles was exported from MedINRIA, which were processed in MATLAB to create binary images of these muscles.

#### 2.2.2. Image segmentation using simpleware ScanIP

The 3D residual limb model was created using Synopsys' Simpleware ScanIP (Synopsys, Mountain View, USA). The binary images that were created by MATLAB, were loaded as background image stacks in ScanIP. These binary image stacks that represent regions of interest (also called masks) are used to create 3D volumetric models. The geometric model for our analysis consisted of the femur, muscle tissues, fat, prosthetic liner and socket, whose construction are described below.

**Muscles**. The muscle masks that were created in the previous section were loaded in Simpleware ScanIP, whose segmentation resulted in volumetric muscle models.

**Femur and socket**. The highly diffused spongiosa, and the relatively low water content in the femur resulted in negligible or random diffusion of water in the DT-MRI scans. This made its segmentation difficult. Furthermore, the socket was also opaque in the DT-MRI scans due to absence of water content. For this reason, the femur and socket were segmented from T1 MP-RAGE sequences (cf. section 2.1).

To register the T1 MP-RAGE sequences with the DT-MRI scans, femurs from both these scans were segmented and exported as surface (STL) models. Using an iterative closest point technique, the transformation that correctly positioned and aligned the surface model of femur segmented from T1 scans (the T1-segmented femur) with that of DT-MRI (the DT-MRI-segmented femur), was determined. This transformation was then applied to the T1-segmented femur and socket, and were imported as CAD models into Simpleware +CAD module.

**Fat**. A mask of the complete residual limb was created from the DT-MRI-DICOMs. A boolean difference of this mask from the union of the muscle and femur masks resulted in the mask for fat tissues.

**Liner**. The subject's liner was 9 mm thick. This liner was modelled by uniformly extending the outer surface of the residual limb in the normal direction towards its exterior by 9 mm.

A Python script automated the process of loading the above masks into Simpleware ScanIP. Morphological and smoothing filters (dilation and recursive Gaussian) were applied on all masks to generate smooth surfaces. After post-processing, the maximum difference between the volumes of muscle masks before and after smoothing was <0.2%.

#### 2.2.3. Generation of the FE model

Using Simpleware ScanIP +FE, an FE mesh of the subject's residual limb was created for LS-DYNA (LSTC, Livermore, California). All parts were meshed with linear tetrahedral elements. The mesh parameters were: coarseness -10, number of quality optimisation cycles 10, mean Jacobian of 0.5 and minimum of 0.1. The parts were allowed to change during meshing, where the maximum distance that a surface node can be displaced off the original surface was limited to 0.2 mm. Contacts were not defined in Simpleware ScanIP, which resulted in common nodes between the tissues in the FE mesh.

**Figure 3:**
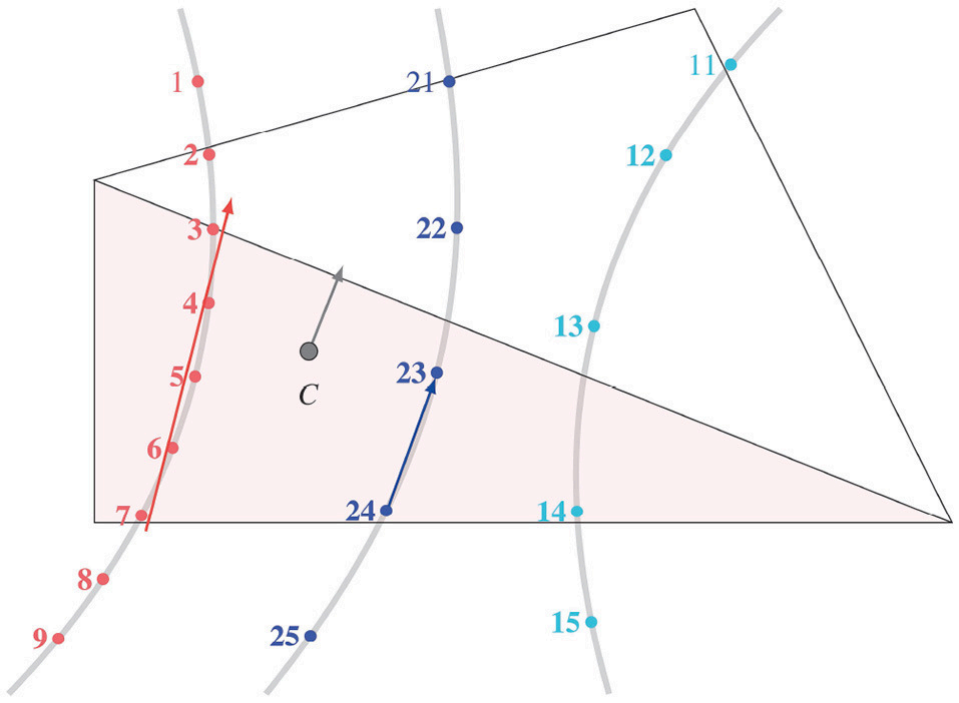
Effective fibre direction in an element. The points within a fibre strand created by MedINRIA were sequentially numbered. When more than one fibre strand passed through an element, the effective fibre direction at the element centroid *C* was computed by the weighted sum of the best fit lines from each strand.

As mentioned earlier in section 1, the muscle fibres were not only meant to automate the modelling process but also to enhance the FE mesh with directional fibre information, which are required in muscle activation studies. The fibres, that were in the NIfTI coordinate system (CS), were aligned with the FE mesh by transforming them back into the DICOM CS. These fibres were not physically modelled and embedded in the FE mesh but rather the fibre anisotropy within each element of the muscles was modelled using the “element_solid_ortho” card in LS-DYNA. To do so, the feature that fibres exported from MedINRIA were consecutively numbered was exploited in determining the number of fibre strands passing through an element. Three fibre strands passing through two elements of the FE mesh are illustrated in Figure 3. The effective fibre direction in an element is the weighted sum of the trend lines fitted for each set of consecutively numbered fibre points in that element. In general, if an FE mesh consists of *N*_*e*_ elements, where $N_{s}^{i}$ fibre strands traverse the *i*^th^ element (*i*∈*N*_*e*_) with its centroid **x**_*i*_, and **g**_*s*_ was the line of best fit for the strand *s* with weight *w*_*s*_, the effective fibre direction in that element, **f**(**x**_*i*_), was defined as

(2) $$\begin{array}{r} \begin{array}{c} \text{f}\left({\text{x}}_{i}\right)=\overset{N_{s}^{i}}{\underset{s=1}{\sum }}w_{s}{\text{g}}_{s},\\ \text{with }w_{s}=\frac{n_{s}^{2}}{\overset{N_{s}^{i}}{\underset{s=1}{\sum }}n_{s}^{2}}, \end{array} \end{array}$$

where *n*_*s*_ was the number of fibre points in the fibre strand *s*. The effective fibre direction in all *N*_*e*_ mesh elements was determined.

**Figure 4:**
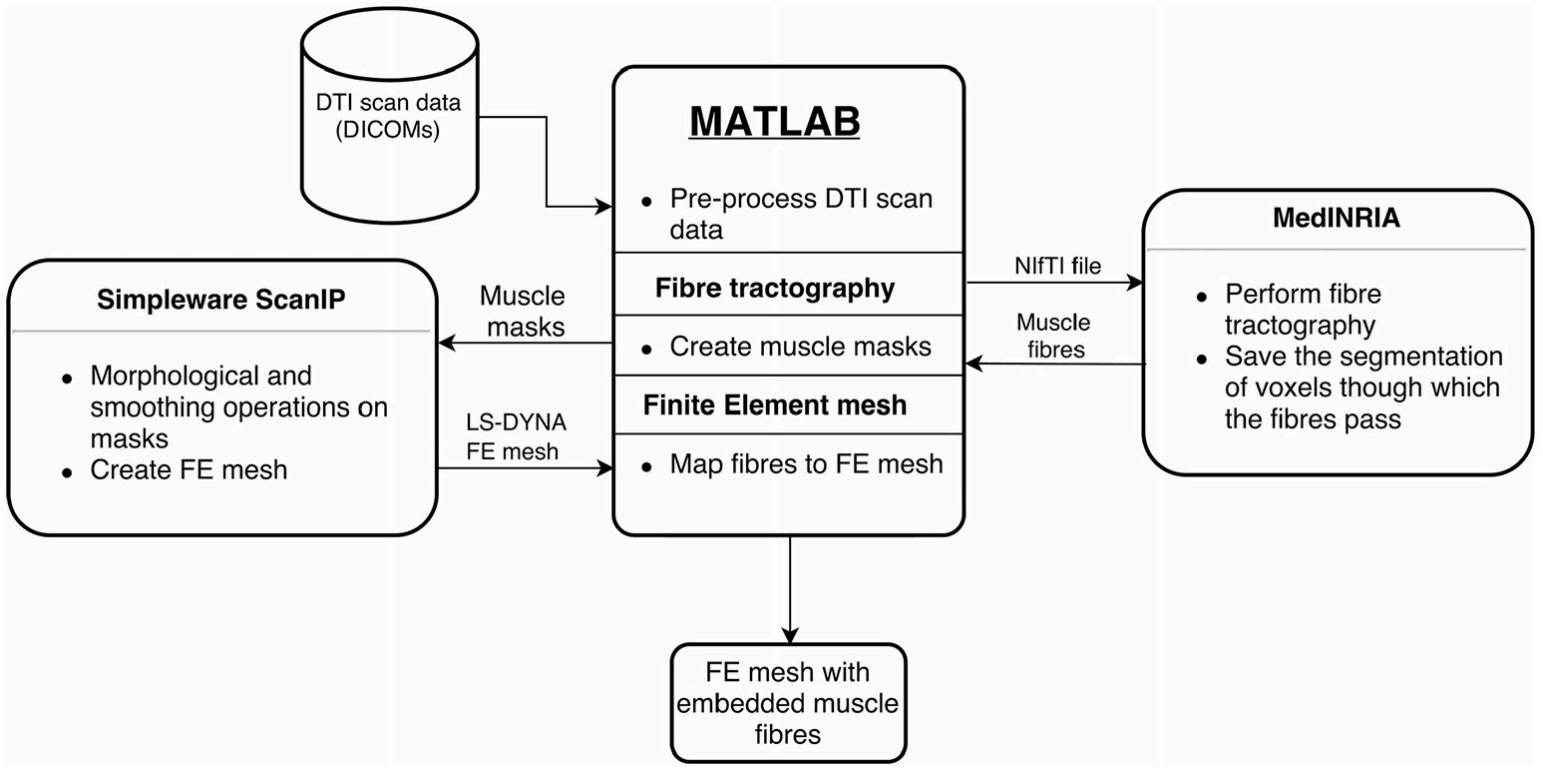
Overview of implemented workflow. The proposed workflow that converts the DT-MRI scans into patient-specific residual limb model is illustrated here. MATLAB handles data processing and transfer between the various software tools. Fibre tracking is done using MedINRIA. Binary muscle masks are created from the voxel segmentation data, which are loaded into Simpleware ScanIP. After image processing in ScanIP, the FE mesh is generated with the ScanIP +FE module. Finally, the extracted fibres are embedded into the FE mesh.

The estimation of effective fibre direction in some elements showed inconsistencies with the fibre directions in their immediate neighbourhood. In some other elements of the mesh, like near the boundary of *Gluteus Maximus*, no fibres were detected. This is attributed to the partial volume effects, and morphological and smoothing filters applied during image processing using Simpleware ScanIP that changed the muscle volume, resulting in missing some fibre information within the boundary elements. To correct the orientation of fibres, and to enhance the model, the fibre field was smoothed using radial basis interpolation with a Gaussian kernel. The new fibre direction in the *i*^th^ element with its centroid at **x**_*i*_, $\tilde{\text{f}}\left({\text{x}}_{i}\right)$, was defined as

(3) $$\begin{array}{r} \begin{array}{c} \tilde{\text{f}}\left({\text{x}}_{i}\right)=\overset{N_{e}}{\underset{e=1}{\sum }}\phi \text{f}\left({\text{x}}_{e}\right)\\ \text{with }\phi =\hat{\phi }\left({\text{x}}_{i},{\text{x}}_{e},p\right)=\exp{\left(-p\frac{\left||{\text{x}}_{i}-{\text{x}}_{e}|\right|}{L}\right)}^{2}, \end{array} \end{array}$$

where **f**(**x**_*e*_) was the original effective fibre direction in the element with centroid at **x**_*e*_ [cf. Equation (2)], ϕ was the Gaussian radial basis function kernel, the scalar parameter *p* set the number of neighbouring elements that influenced the fibre orientation in the current element (here, *p* = 20), and *L* was the length of the diagonal of the bounding box formed by mesh elements of the muscle. The above steps were performed for all 11 muscles in the residual limb. This resulted in the final FE mesh for our analyses. An overview of the entire workflow is shown in Figure 4.

### 2.3. Constitutive model for soft tissues

Skeletal muscle contractions occur as a result of chemical interactions resulting in physical movement of muscle filaments. The grand sum of individual filament contractions results in the overall muscle contraction. These muscle contractions can be described in varying levels of detail (see Heidlauf and Röhrle, 2014; Bradley et al., 2018, for more details). The continuum-mechanical model for the soft tissues, within this work, was based on a 3D phenomenological approach, i.e., the constitutive model of the muscles is based on macroscopically observed muscle contractions as presented in Röhrle et al. (2017).

It is assumed that the force developed by the muscle fibres is additively split into an active and a passive part. Passive force is generated when fibres stretch, while an active force is related to muscle contraction stemming from chemo-electro-mechanical processes on the cellular level. In order to model these processes in a continuum-mechanical sense, these contributions were expressed as a function of the fibre's length or stretch. Time-dependent viscoelastic behaviour of soft tissues was ignored, and the soft tissues were modelled as hyperelastic, quasi-incompressible materials. Muscles are assumed to undergo large deformations, and therefore, the theory of finite elasticity is assumed. The material model for soft tissues implemented here is based on Röhrle et al. (2017). In the following, a brief overview of the theory and constitutive equations for the material model are given.

#### 2.3.1. Constitutive muscle model

A continuum body B is assumed to be a collection of points P in space whose local deformation can be described by the deformation gradient **F**. The deformation gradient **F** is a map between the reference and the current configuration, and is defined as **F** = **Grad x** = ∂_****X****_**x**, where **X** is the position vector to a material point P at the reference configuration, and **x** is the position vector to the same point in the deformed configuration of the body B. In the theory of Finite Elasticity, the deformed state of the body B is formulated in terms of the right or the left Cauchy-Green tensor, which are defined as **C** = **F**^T^**F**, and **B** = **FF**^T^, respectively. The balance of linear momentum is given by

(4) $$\begin{array}{r} \text{div}\text{T}+\rho \text{g}=\rho \ddot{\text{x}},\;\text{with}\rho =\frac{1}{\text{det}\text{F}}\rho_{0}, \end{array}$$

where **T** is the Cauchy stress, ρ_0_ is the material density in the reference configuration, ρ is the density in the current configuration, **g** is the acceleration due to gravity, and $\ddot{\text{x}}$ is the acceleration of spatial points P∈B.

The stress developed in the soft tissues is additively split into one part stemming from the isotropic matrix and one part originating from anisotropic behaviour associated with the fibre direction. Likewise, the stress in the fibre direction is additively split into its active and passive contributions. Therefore, the second Piola-Kirchhoff stress tensor, **S**, can be expressed as

(5) $$\begin{array}{r} \text{S}={\text{S}}_{\text{iso}}+{\text{S}}_{\text{aniso}}={\text{S}}_{\text{iso}}+\left(1-\gamma \right)\left({\text{S}}_{\text{pas}}+\alpha {\text{S}}_{\text{act}}\right), \end{array}$$

where **S**_iso_ and **S**_aniso_ are the isotropic and anisotropic parts of the stress tensor. Likewise, the passive and active contributions to the anisotropic stress tensor are **S**_pas_ and **S**_act_. As mentioned earlier (cf. section 2.3), the material model developed here was meant to be applicable for soft tissues, i.e., fat, skeletal muscles and tendons. For this reason, a binary variable γ was introduced to control the exclusion or inclusion of the stress contribution from fibres. For example, γ = 1 results in purely isotropic stress contribution, while γ = 0 allows one to consider the anisotropic contributions due to the fibres as well. Muscle activation is given by α, with 0 ≤ α ≤ 1.

The strain energy formulation for the compressible isotropic soft tissue matrix was taken from Crisfield (1997). The isotropic second Piola-Kirchhoff tensor is

(6) $$\begin{array}{r} {\text{S}}_{\text{iso}}=\left(B_{1}\text{I}+B_{2}\text{C}+B_{3}{\text{C}}^{-1}\right)+k\left(\text{det}\text{F}-1\right){I_{3}}^{1/2}{\text{C}}^{-1}, \end{array}$$

where *k* is the bulk modulus of the soft tissue matrix, and

(7) $$\begin{array}{r} \begin{array}{c} B_{1}=2C_{1}I_{3}^{-1/3}+2C_{2}I_{3}^{-2/3}I_{1},\\ B_{2}=-2C_{2}I_{3}^{-2/3},\;\text{and}\\ B_{3}=-2/3C_{1}I_{3}^{-1/3}I_{1}-4/3C_{2}I_{3}^{-2/3}I_{2}. \end{array} \end{array}$$

Here, *C*_1_ and *C*_2_ are material parameters of the isotropic part of the strain energy function, and *I*_1_, *I*_2_ and *I*_3_ are the invariants of **C**. The fourth invariant of the transversely isotropic material is

(8) $$\begin{array}{r} \begin{array}{c} I_{4}\left(\text{C},\text{M}\right)=\text{M}:\text{C},\\ \text{where }\text{M}={\text{a}}_{0}⊗{\text{a}}_{0}, \end{array} \end{array}$$

where **M** is a structural tensor defined by means of the fibre direction, **a**_0_ in the reference configuration. Then, one can define the fibre stretch Λ as $\Lambda =\sqrt{I_{4}}$.

The active and passive contributions of fibres to the second Piola-Kirchhoff stress (cf. Röhrle et al., 2017) are

(9) $$\begin{array}{c} S_{\text{aniso}}=\left(1-\gamma \right)\left(S_{\text{pas}}+\alpha S_{\text{act}}\right),\text{ where}\\ S_{\text{pas}}=\{\begin{array}{ll} \frac{1}{\Lambda^{2}}C_{3}\left(\Lambda^{C_{4}}-1\right)\;M, & \text{if }\Lambda \geq 1,\\ 0, & \text{otherwise},\text{ and} \end{array}\\ S_{\text{act}}=\{\begin{array}{l} \frac{S_{\text{max}}}{\Lambda^{2}}\exp\left(-|\frac{\Lambda /\Lambda_{\text{opt}}-1}{\Delta W_{\text{asc}}}\;{|}^{\nu_{\text{asc}}}\right)\;M,\text{ if }\Lambda \leq \Lambda_{\text{opt}},\\ \frac{S_{\text{max}}}{\Lambda^{2}}\exp\left(-|\frac{\Lambda /\Lambda_{\text{opt}}-1}{\Delta W_{\text{dsc}}}{|}^{\nu_{\text{dsc}}}\right)\;M,\text{ if }\Lambda >\Lambda_{\text{opt}}. \end{array} \end{array}$$

Here, *C*_3_ and *C*_4_ are material parameters and *S*_max_ is the maximum stress that a muscle can produce at its optimal length (Λ_opt_). The parameters Δ*W*_asc_, ν_asc_, Δ*W*_dsc_ and ν_dsc_ affect the magnitude of the active part of the Piola-Kirchhoff stress. The material parameters chosen for the muscles are given in Table 1. The material tangent, ℂ, that is required for implicit computations is a fourth-order tensor obtained from the second-order Piola-Kirchhoff stress tensor as ℂ = ℂ_*MNOP*_ = 2∂_**C**_**S**. It is noted that the implementation within LS-DYNA requires the definition of the spatial counterpart of ℂ, which is denoted by *B*. But *B* is easily obtained by the push-forward of the material tangent ℂ, i.e.,

(10) $$\begin{array}{r} B=J^{-1}\chi_{*}\left(\mathbb{C}\right)={\left(\text{F}⊗\text{F}\right)}^{\overset{23}{\text{T}}}\mathbb{C}{\left({\text{F}}^{\text{T}}⊗{\text{F}}^{\text{T}}\right)}^{\overset{23}{\text{T}}}. \end{array}$$

The superscript 23 above the transpose indicates that the transposition is defined by the exchange of second and third bases in the dyadic product. Hence, the spatial elasticity tensor is given in index notation by

(11) $$\begin{array}{r} B_{ijkl}=F_{iM}F_{jN}F_{kO}F_{lP}{\mathbb{C}}_{MNOP}. \end{array}$$

#### 2.3.2. Tissue injury model

Exposing the residual limb to excessive stresses and strains leads to soft tissue damage through cell death (see Oomens et al., 2014). This is also associated with deep tissue injury, whose timely prognosis is difficult. Using patient-specific limb models, FE analyses can provide the internal stress state of soft tissues; the basis for predicting tissue damage.

**Table 1:** Material parameter table.

**Model**	**Material parameter**	**Contribution**	**Value**
Muscle (Röhrle et al., 2017)	$C_{1}^{\text{M}}$	Isotropic	2.5 × 10^−6^ MPa
	$C_{2}^{\text{M}}$	Isotropic	6 × 10^−3^ MPa
	$C_{3}^{\text{M}}$	Anisotropic (passive)	1 × 10^−3^ MPa
	$C_{4}^{\text{M}}$	Anisotropic (passive)	6 (–)
	*S*_max_	Anisotropic (active)	0.1 MPa
	Δ*W*_asc_	Anisotropic (active)	0.15 (–)
	Δ*W*_dsc_	Anisotropic (active)	0.16 (–)
	ν_asc_	Anisotropic (active)	2 (–)
	ν_dsc_	Anisotropic (active)	4 (–)
	Λ_opt_	Anisotropic (active)	1.3 (–)
	γ^M^	-	0
	α^M^	-	0
Skin/Fat (Röhrle et al., 2017)	$C_{1}^{\text{S}}$	Isotropic	2.5 × 10^−6^ MPa
	$C_{2}^{\text{S}}$	Isotropic	6 × 10^−3^ MPa
	$C_{3}^{\text{S}}$	Anisotropic (passive)	1 × 10^−3^ MPa
	$C_{4}^{\text{S}}$	Anisotropic (passive)	6 (–)
	*S*_max_	Anisotropic (active)	0.1 MPa
	Δ*W*_asc_	Anisotropic (active)	0.15 (–)
	Δ*W*_dsc_	Anisotropic (active)	0.16 (–)
	ν_asc_	Anisotropic (active)	2 (–)
	ν_dsc_	Anisotropic (active)	4 (–)
	Λ_opt_	Anisotropic (active)	1.3 (–)
	γ^S^	-	1
	α^S^	-	0
Liner (Łagan and Liber-Kneć, 2018)	$C_{1}^{\text{L}}$	Isotropic	0.33 MPa
	$C_{2}^{\text{L}}$	Isotropic	0.01 MPa
Femur (Zhang et al., 2013)	ρ (density)	-	1 × 10^−3^ g·mm^−3^
	*E* (Young's modulus)	-	1.5 × 10^4^ MPa
	ν (Poisson ratio)	-	0.27
Socket (Zhang et al., 2013)	ρ (density)	-	1 × 10^−3^ g·mm^−3^
	*E* (Young's modulus)	-	1.0 × 10^4^ MPa
	ν (Poisson ratio)	-	0.30

*Parameters for the material model. Parameters for muscle and Skin/Fat were adapted from Röhrle et al. (2017)*.

The main idea of Gefen et al. (2008) is that cell death occurs if ε_eff_ > ε_crit_, where ε_eff_ is the effective strain in the cell, and ε_crit_ is the critical strain limit beyond which cell death occurred. The effective and critical strains are defined as

(12) $$\begin{array}{r} \begin{array}{c} {\text{ε}}_{\text{eff}}=\sqrt{\frac{2}{3}\varepsilon_{ij}\varepsilon_{ij}},\;\text{and}\\ \varepsilon_{\text{crit}}=\frac{K}{1+\exp\left(\beta \left(t-t_{0}\right)\right)}+C, \end{array} \end{array}$$

where **ε** is the symmetric Green strain. The values for the empirical constants in Equation (12) are *K* = 0.268, $t_{0}\;\text{(time of midpoint of the sigmoid step)}=9.78\times 10^{6}\;\text{ms}$, β = 5.83 × 10^−7^ms^-1^ and *C* = 0.332 (see Gefen et al., 2008). The Boltzmann-type sigmoid curve seen in Equation (12) provides the evolution of critical strain based on which a cell was identified as either injured or healthy. This injury model was implemented in the material model.

The material parameters of the soft tissues, bone, liner and socket used in the FE simulations are provided in Table 1.

### 2.4. Loading and boundary conditions for FE analysis of bipedal stance

The dynamic FE analysis of bipedal stance is performed in two stages: socket donning, followed by constant femoral load over a period of 2 h. The boundary conditions, loads and contact definitions applied in the analysis are detailed here.

For the generated FE mesh of the residual limb, no contact pairs were defined (cf. section 2.2.3). This resulted in common nodes between adjacent parts of the residual limb (muscles, bone, fat, liner) at their boundaries. A frictionless surface-to-surface mortar contact was defined between the outer surface of the liner and inner surface of the socket.

**Figure 5:**
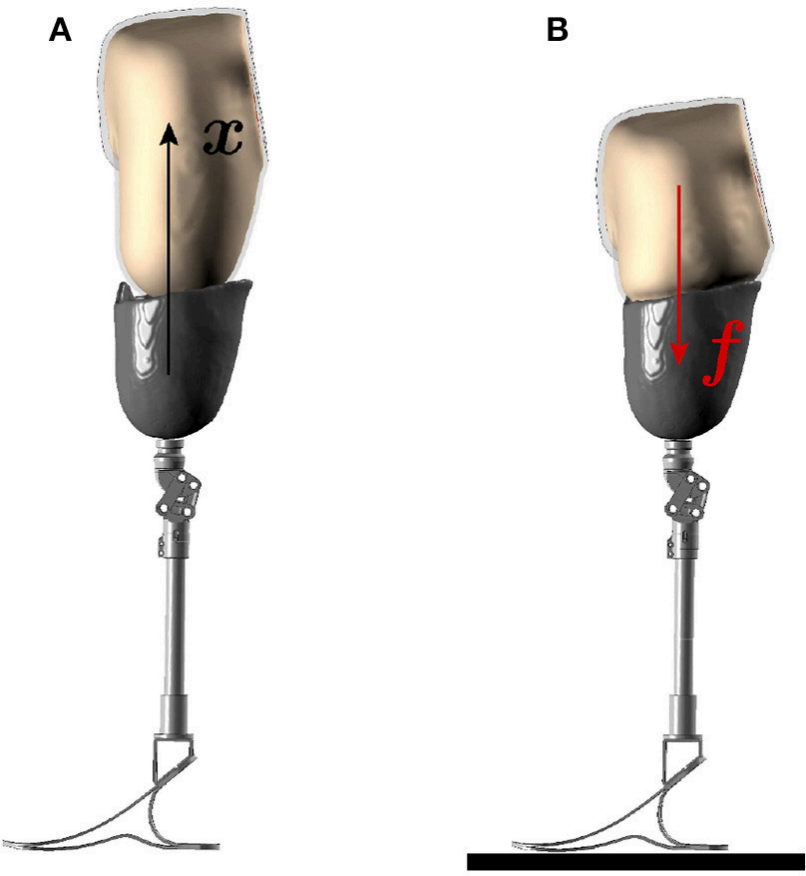
Boundary conditions for FE analysis. The two stages of the socket donning analysis are illustrated here. **(A)** In the first stage, the socket is donned over the residual limb through prescribed motion boundary condition. **(B)** In the next stage, the patient's body weight is applied on the femur in the socket-donned state as the prosthesis rests on the ground.

For the FE analysis of bipedal stance, the residual limb and prosthesis were only permitted to translate in the craniocaudal axis, while the other 5 degrees of freedom (DoF) were constrained. In the initial state, the socket and residual limb-liner complex were separated by a distance of 130 mm in the craniocaudal direction. In the socket donning simulation, the femur was constrained in all DoFs and the socket was translated towards the limb (superior) by 130 mm along the craniocaudal axis in 10 s (see also Figure 5A). At the end of the socket donning simulation, the nodes of the liner that were in contact with the socket, were tied to the socket using tied-nodes-to-surface penalty-based contact in LS-DYNA. The translational constraint along the craniocaudal axis that was imposed on the femur during the socket donning simulation was removed. This was followed by the application of 400 N femoral load, which is approximately half the subject's body weight, acting in the positive inferior direction for 2 h (see also Figure 5B). This duration (2 h) was selected to be long enough to study strain-time dependent cell death.

The residual limb, socket and liner were meshed with 1-point, constant pressure, solid tetrahedral elements. The dynamic, implicit analysis was solved using Davidon-Fletcher-Powell (DFP) quasi-Newton iterative solver with LS-DYNA on a Linux workstation with 4 processors [Intel(R) Xeon(R) CPU E5-2687W, 3.10 GHz] using 1.12 GB RAM.

## 3. Results

In the following, we present the outcome of the proposed modelling-simulation-analysis workflow, i.e., the result of the geometry and FE mesh generation of the residual limb, and how this model can be used to analyse and compare stresses and injured volumes between fused- and individual-muscle models in the socket-donned state, with the original and misfitted sockets, during bipedal stance.

### 3.1. Geometry and FE mesh of the residuum

**Figure 6:**
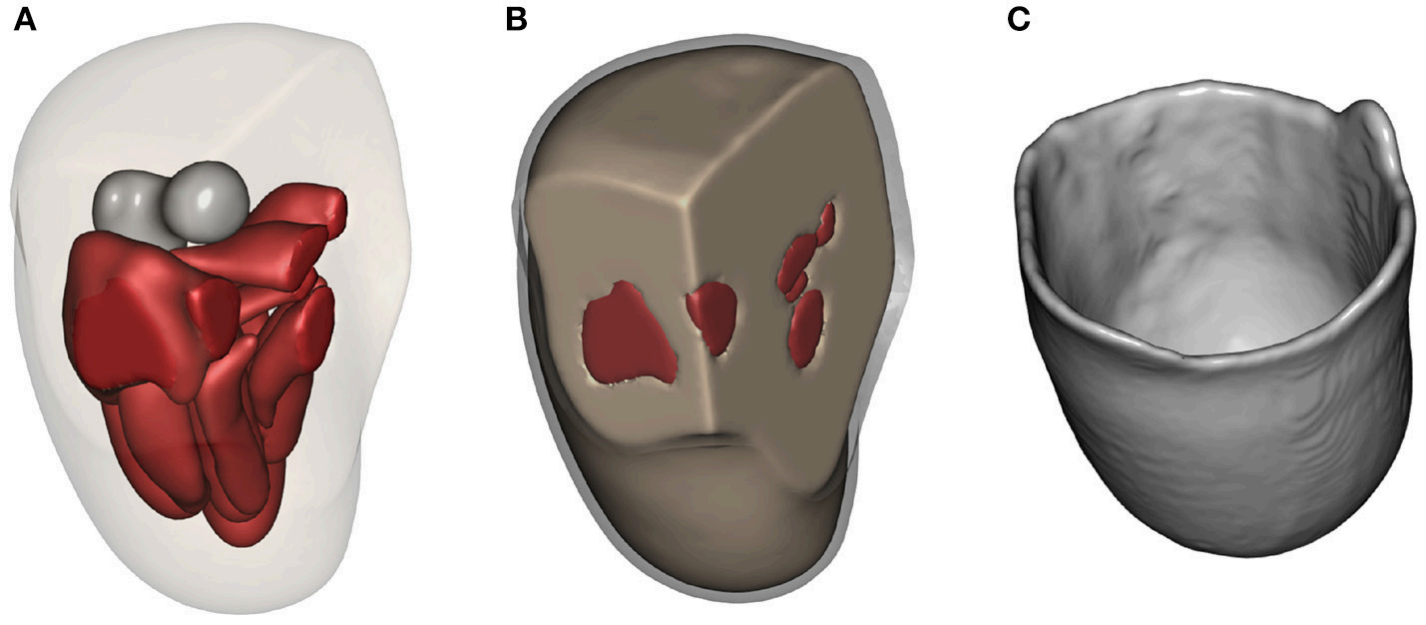
Residual limb model. **(A)** The residual limb model created in Simpleware ScanIP. The opacity of liner and fat layers have been reduced to reveal the underlying bone and muscles. **(B)** The liner around fat/skin layer is shown in grey. **(C)** Model of the original socket worn by the subject.

**Figure 7:**
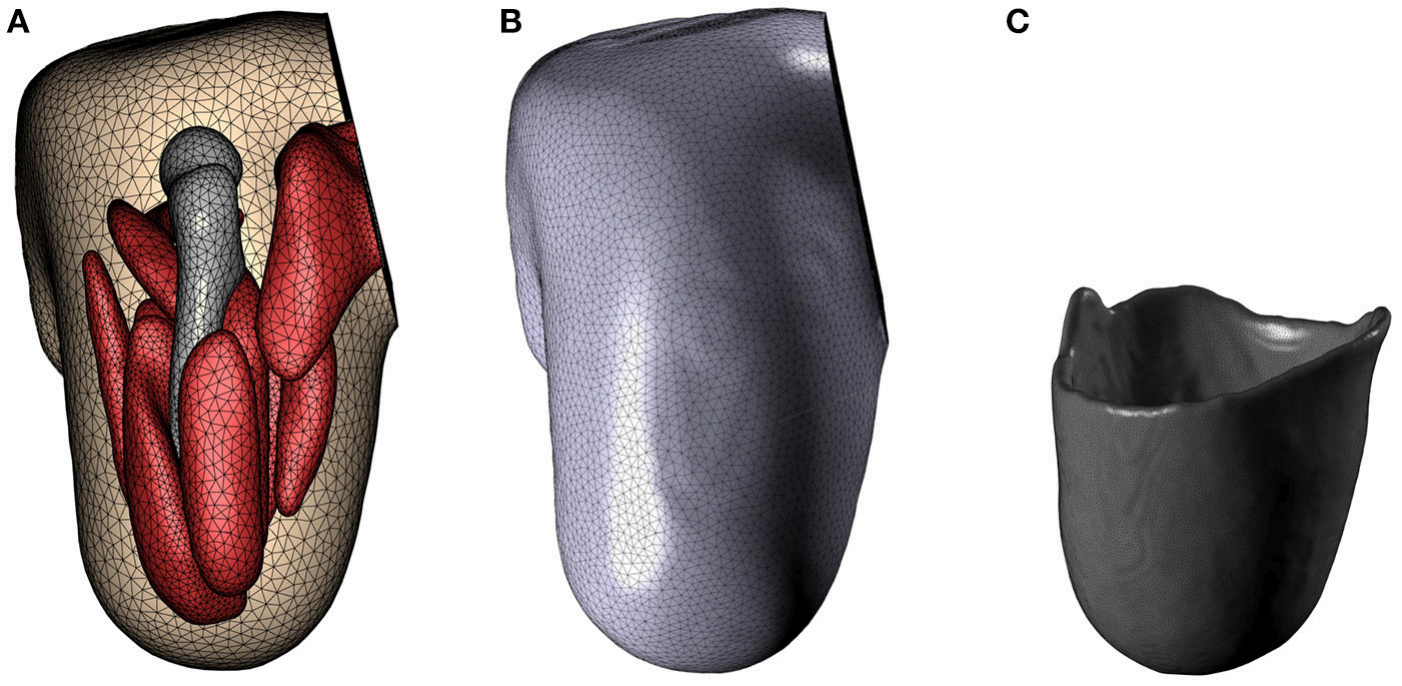
FE mesh. FE meshes for the residual limb, liner and socket are presented in **(A–C)**, respectively.

The results of applying the above described modelling workflow to the imaging data results in the discretised residual limb model, which is shown in Figure 6. Figure 6A depicts the geometries of the bone (residual femur) and the skeletal muscles within the stump by suppressing the opacity of the outer skin and liner while Figure 6B shows the stump including the fat/skin and liner geometry, and Figure 6C presents the model of the original socket worn by the subject. The FE meshes of these models, constructed with the mesh parameters listed in section 2.2.3, are shown in Figure 7. In Figure 7A, the FE mesh of muscles and bone is superimposed on the mesh of the skin. The linear Lagrange tetrahedral FE meshes of liner and socket are shown in Figures 7B,C. The mesh consisted of 5, 416 elements in the femur, 35, 257 elements in the muscles, 97, 503 elements in the skin/fat layer, 51, 791 elements in the liner, and 327, 865 elements in the socket.

**Figure 8:**
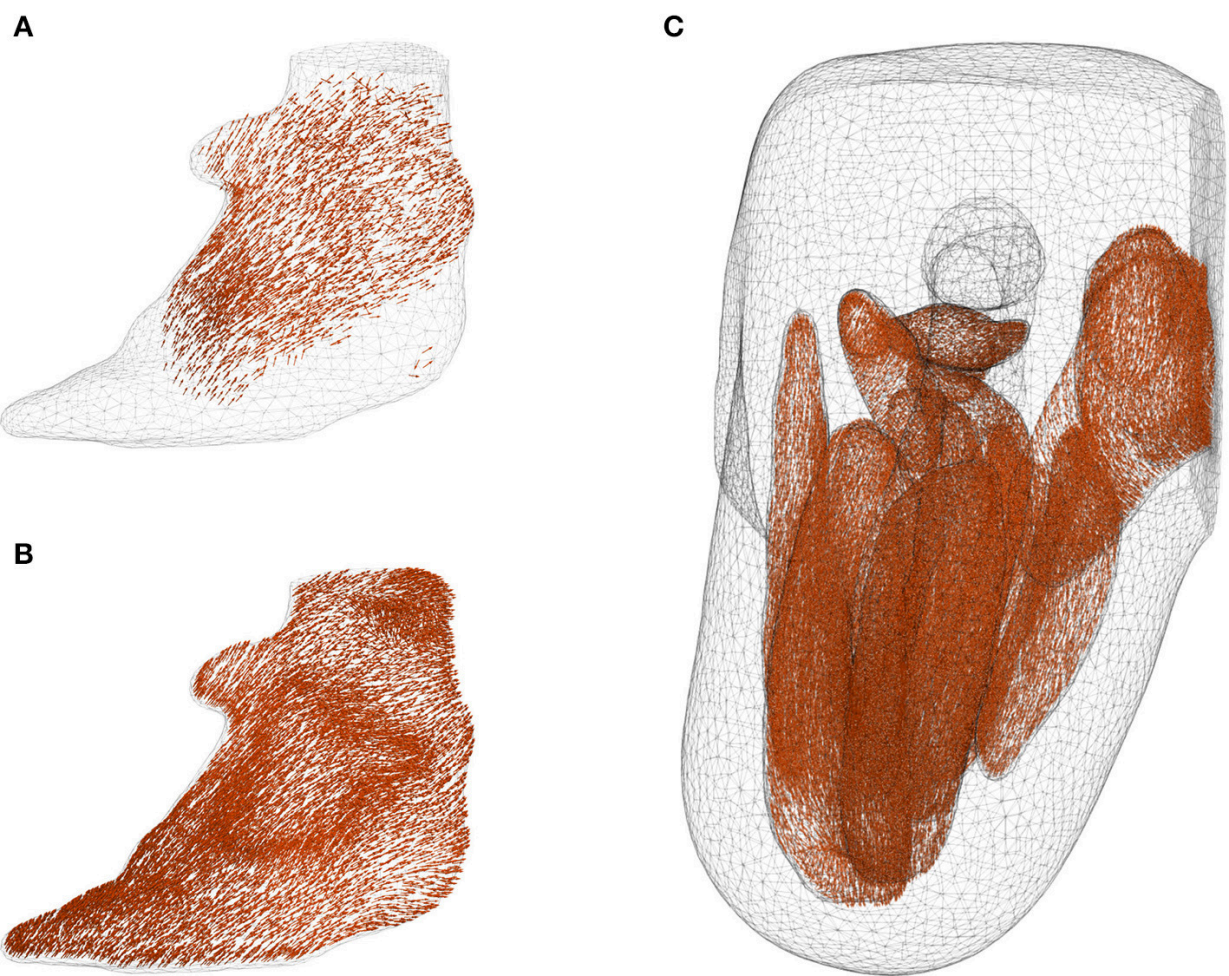
Corrected fibre orientation. **(A,B)** show the fibres in *Gluteus Maximus* before and after interpolation using radial basis function, respectively. It can be seen that some boundary elements in **(A)** contained no fibre information. The inconsistent and missing fibres were corrected through the interpolation method proposed in section 2.2.3, and the resulting fibre distribution is shown in figure **(B)**. Fibre orientation in the muscles are shown in **(C)** with femur, fat and liner suppressed for the sake of clarity.

The fibres that were mapped from MedINRIA into the FE mesh suffered from inconsistent fibre directions among neighbouring elements, and with some elements completely devoid of fibre information. This is shown in the case of *Gluteus Maximus* in Figure 8A. With the fibre-mapping algorithm described in section 2.2.3, these inconsistencies were corrected, which resulted in the fibre distribution in the *Gluteus Maximus* as shown in Figure 8B. The direction of muscle fibres in the complete residual limb is shown in Figure 8C.

The duration of the overall modelling process, from segmenting the DT-MRI scans to creating the FE model of the residual limb with tissue anisotropy, was about 20 min.

### 3.2. Mesh convergence studies

**Figure 9:**
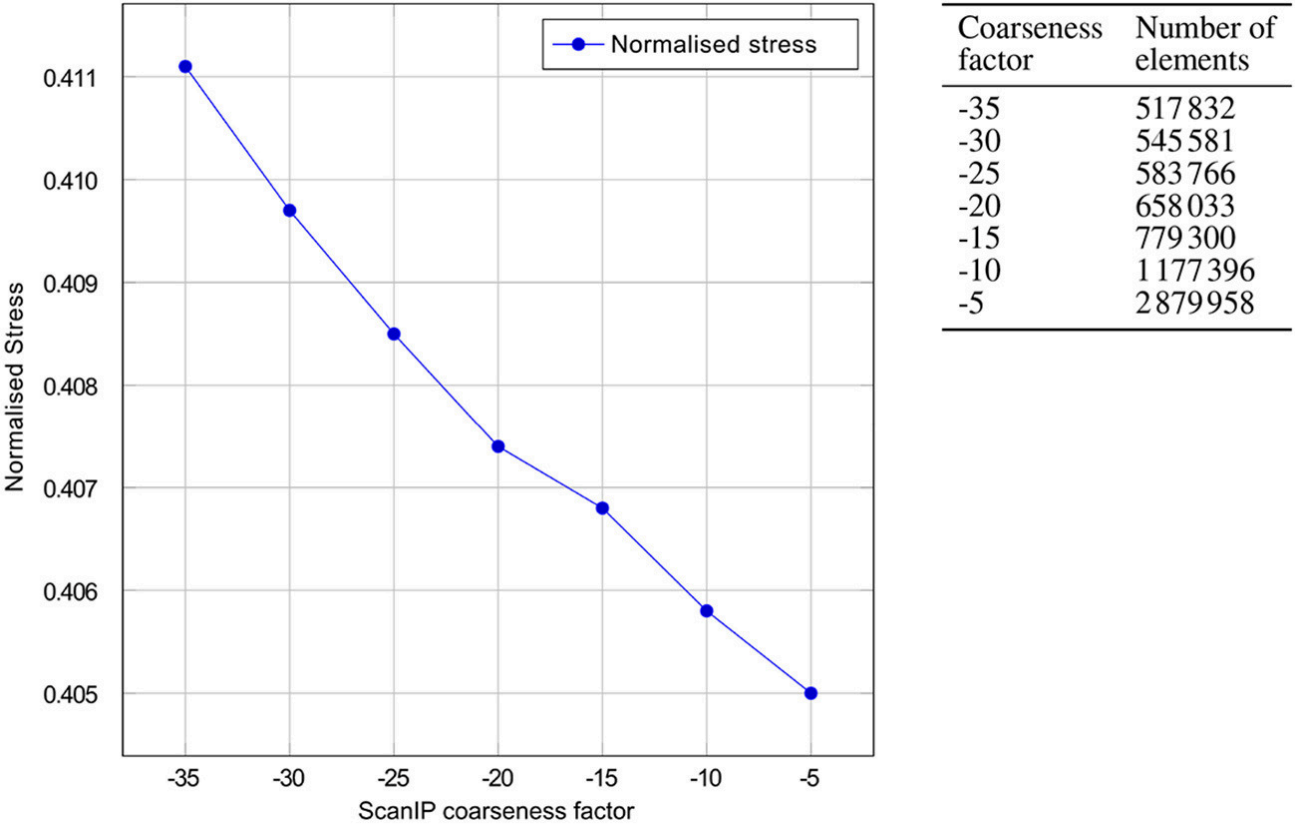
Mesh convergence. Convergence of the normalised von Mises stresses of the residual limb model with decreasing ScanIP coarseness factor is shown. On the right, the number of linear tetrahedral elements in the FE model is tabulated against the ScanIP coarseness factors. The graph depicts the decrease in normalised stress with increase of mesh density, i.e., decreasing the mesh coarsening factor from −35 to −5.

Prior to performing the bipedal stance analysis, a mesh convergence study with the boundary conditions described in section 2.4 was performed. Varying mesh densities of the residual limb model were obtained by adjusting the mesh coarseness slider in Simpleware ScanIP +FE between −35 and −5. The selection criteria was based on convergence of volume-normalised stresses in the residuum, with which the optimal mesh was selected. The volume-normalised stress $\bar{\sigma }$ is computed by

(13) $$\begin{array}{r} \bar{\sigma }=\frac{1}{V}\overset{N_{e}}{\underset{e=1}{\sum }}\sigma_{e}v_{e}, \end{array}$$

where σ_*e*_ is the von Mises stress in an element of volume *v*_*e*_, *V* is the total volume of residual limb and *N*_*e*_ is the total number of elements in the residuum. The mesh convergence plot and the mesh densities of the residual limb model are shown in Figure 9. From the graph, one can see that the normalised stress is decreasing with increasing mesh density, in a negligible fashion, i.e., by <1.5%. As a result, the FE model created with coarseness factor of –35 resulting in a mesh with 517, 832 elements was chosen for this study.

### 3.3. Bipedal stance simulation

**Figure 10:**
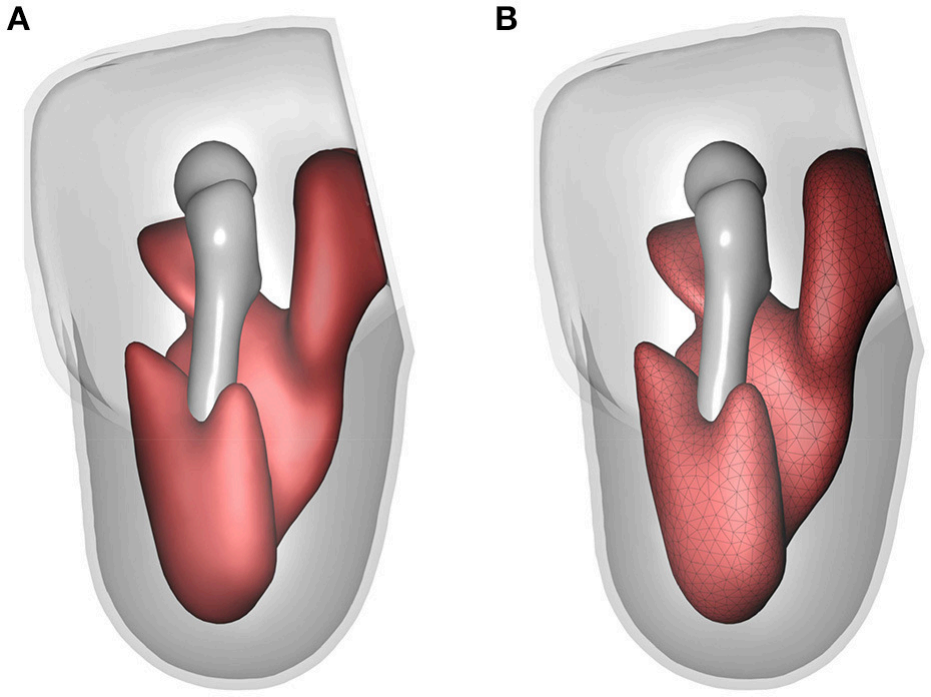
Fused-muscle model. The geometry and FE mesh of the fused muscle are shown in **(A,B)**, respectively.

For highlighting the full capabilities of the modelling-simulation-analysis workflow and for emphasising the need of detailed, individual-muscle models, two models were created. These are (i) the fused-muscle model for the sake of comparison with the proposed individual-muscle model and (ii) a misfitting socket to predict tissue injury. To create the fused-muscle model, masks of individual muscles were fused. Using the same mesh parameters, the FE mesh of the fused-muscle model was generated. The resulting model and the FE mesh are shown in Figure 10. The number of linear tetrahedral elements in the fused muscle model were 477, 989. The misfitting socket was generated by isotropically shrinking the original socket by 10%. This scaling factor is not motivated by any particular application.

**Figure 11:**
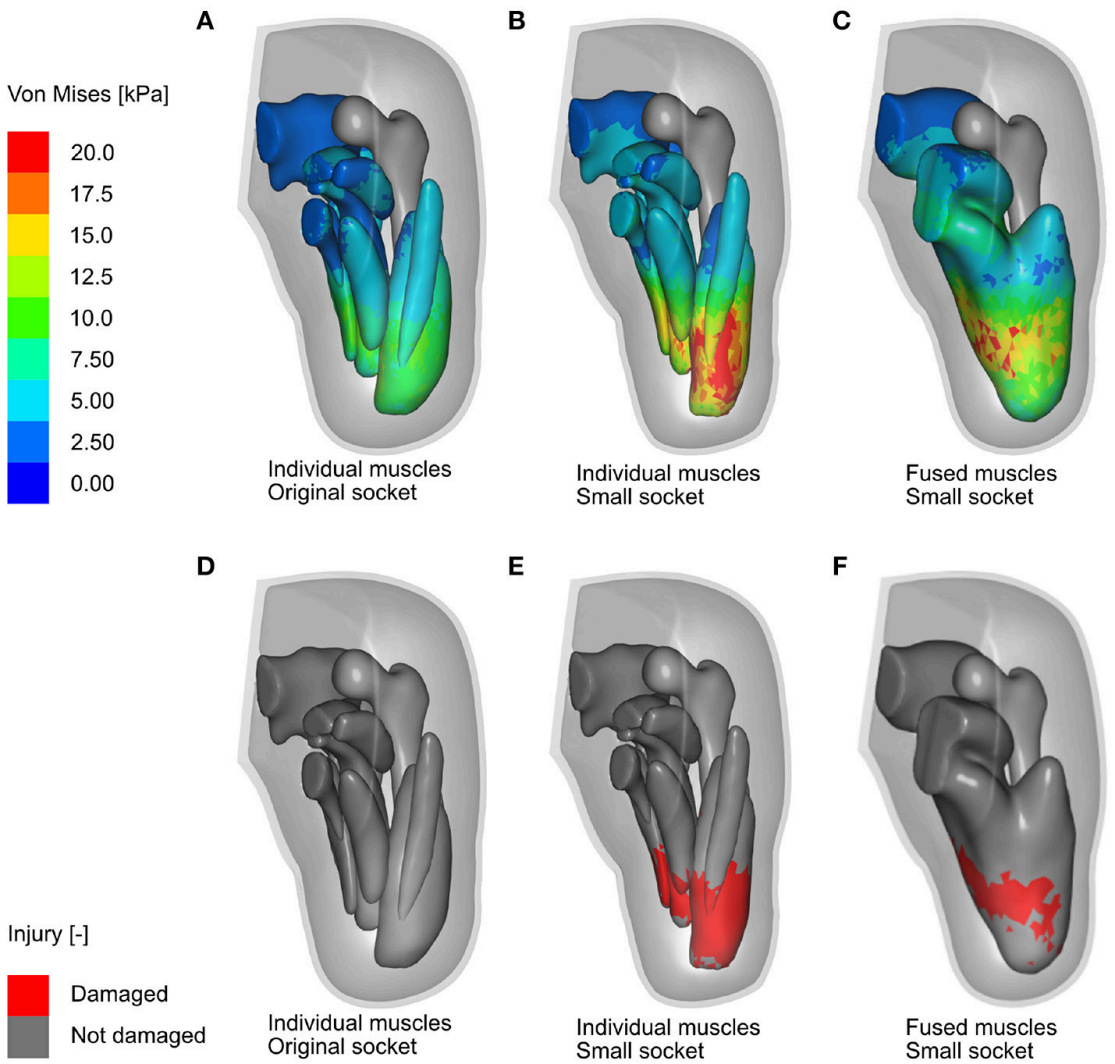
FE analysis of bipedal stance. Shown here are the results of the FE analysis (in the anterior-medial viewing direction) for the three cases discussed above. **(A,B)** Show the Von Mises stresses in the residual limb muscles after the 2-h bipedal stance, when analysing the individual-muscle model of the residual limb with the original and misfitted sockets, respectively, while **(D,E)** Show damage in the skeletal muscles in the same period. **(C,F)** Show the Von Mises stresses and soft tissue damage, respectively, in the fused-muscle model of the residual limb. The stress legend is common for all three cases and was capped at 20 kPa for better illustrating the stress zones.

The bipedal stance simulation was performed using three different limb models, namely (i) the individual-muscle model together with original socket, (ii) the individual-muscle model together with smaller, misfitting socket, and (iii) the fused-muscle model together with the misfitting socket. The von Mises stresses and tissue damage in the residual limb during the bipedal stance analysis are shown for each of the cases in Figure 11. A maximal von Mises stress of 63.5 kPa developed in the individual-muscle model with the misfitting socket, i.e., in case (ii). The peak stresses for cases (i) and (iii) were 19 and 14.34 kPa, respectively.

**Figure 12:**
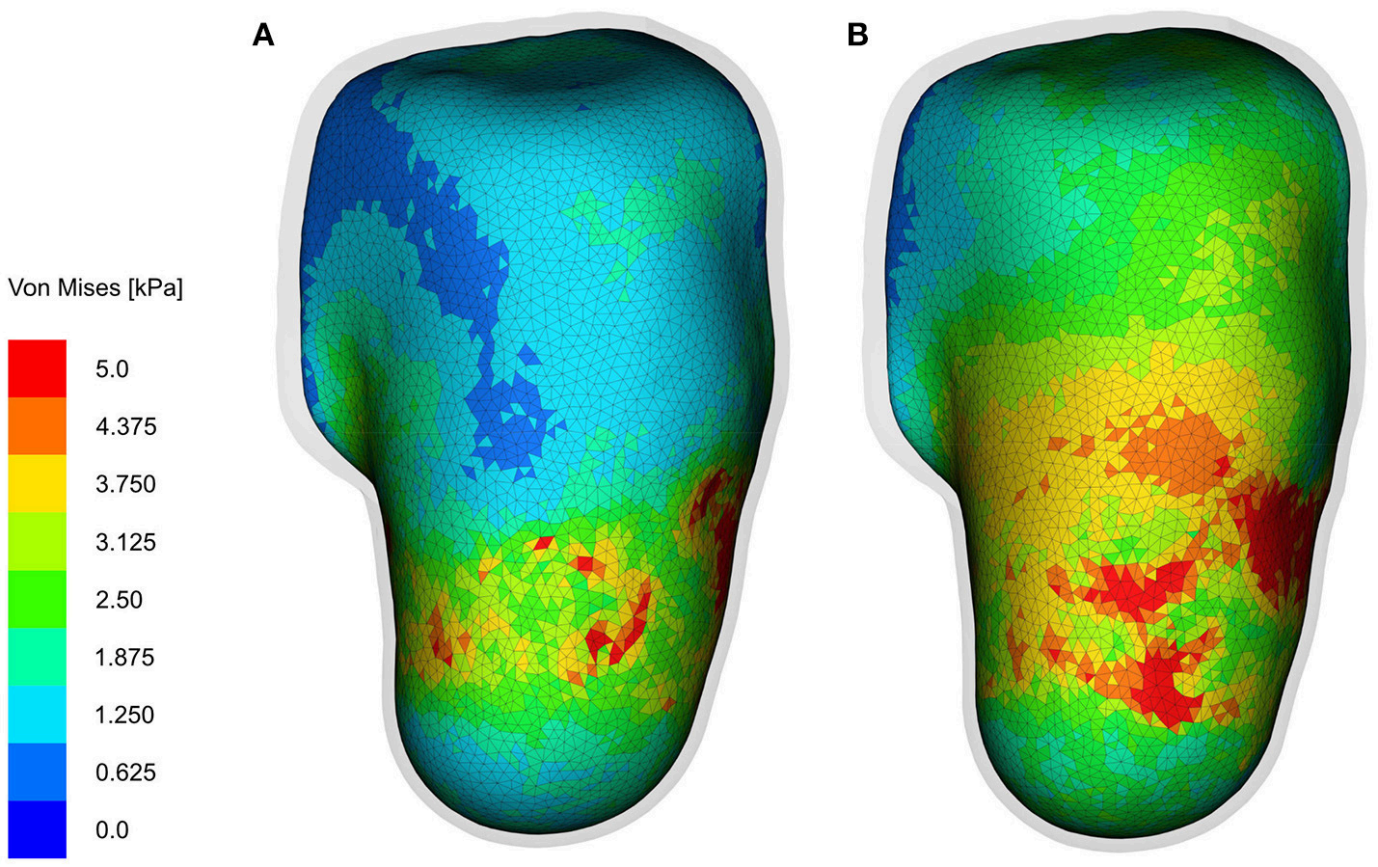
Interface stresses. The interface stresses on the skin/fat layer at the end of 2-h bipedal stance analysis are shown (in the anterio-posterior viewing direction) here. **(A,B)** Show the Von Mises stresses in the fused- and individual-muscle models, respectively. For the sake of clarity, the socket is hidden, and the liner is rendered transparent to show the stresses on the underlying skin/fat layer. The stress legend is common for both the models, and is capped at 5 kPa to better illustrate the stress zones.

**Figure 13:**
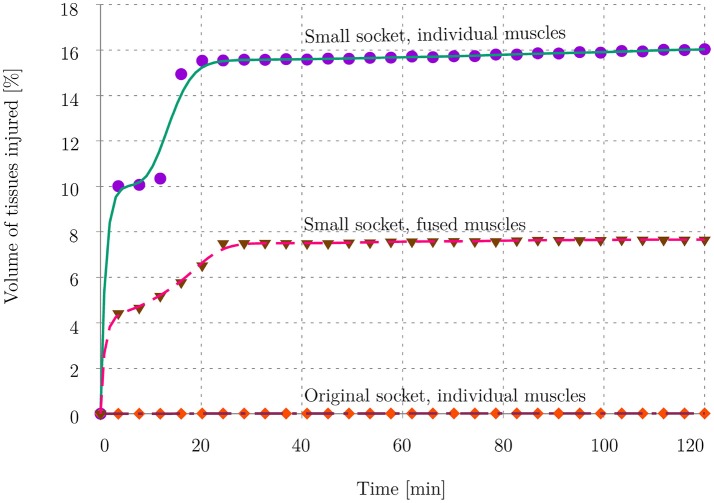
Volume ratio of injured tissues. The plot shows the volume of muscle tissues that was affected by deep tissue injury as a percentage of total muscle volume in the residual limb. The evolution of tissue damage for all 3 cases is plotted here.

Figures 11D–F depicts, for all three cases, the damaged muscle tissues in red. The magnitude of soft-tissue damage for the three cases is plotted in Figure 13. Here, the volume percent of damaged tissues with respect to the total, undamaged volume of the tissues is plotted over the duration of the entire stance phase. It is observed that with the original socket, the volume percent of damage is negligible. The maximum observed damage at the end of the simulation was 0.01%. When simulated with the misfitting socket, i.e., cases (ii) and (iii), this rose to 16.03 and 7.65%, respectively.

The interface stresses on the skin/fat layer in both the fused- and individual-muscle models after the 2-h bipedal stance when donned with the original sockets are shown in Figure 12. The mean and standard deviation of the interface stresses in the fused- and individual-muscle models were 5.4±1.6 and 4.9±1.1 kPa, respectively. The peak stresses in the two models were 16.3 and 24.1 kPa, respectively, which were observed in the proximal anterior and posterior regions of the residual limb.

## 4. Discussion

In this study, we proposed an efficient modelling-simulation-analysis workflow to investigate stump-socket interaction during bipedal stance and hypothesised that detailed, individual-muscle models of residual limbs will provide more insight into the prosthesis-stump interaction than that provided by fused-muscle models. This study can be considered as a proof of concept for future work on automating and optimising socket design from imaging data.

### 4.1. DT-MRI-to-FE mesh workflow

The core of the modelling-simulation-analysis workflow of this research is the quasi-automatic generation of detailed patient-specific FE models of residual limbs from DT-MRI scans. The model generation relies on the fibres extracted from DT-MRI scans, i.e., the muscle and other soft tissue masks that are imported to Simpleware ScanIP, are created from the tracked fibres. But the process of extracting these fibres is done manually, and therefore subjected to some degree of human-related error. For example, the influence of parameters set in MedINRIA's DTI Track module, in the resulting fibre distribution, and in the muscle masks that were generated, is not known. It is expected that changes in these parameters might affect the volume of muscle masks but not the resulting fibre orientations. Another potential source of error is due to the manual grouping of muscle fibres into bundles, in MedINRIA. This can lead to some fibres being simultaneously grouped into two muscle bundles, and therefore result in overlapping muscle masks in Simpleware ScanIP, where the stacking order of these masks plays a vital role in determining the resulting model and FE mesh. In case of overlapping volumes, the masks at the top of the stack overwrite the volume of intersecting masks below them. Hence, different models can result from different mask hierarchies. Moreover, smoothing and morphological operations in ScanIP, which are essential to obtain a smooth and kink-free FE mesh, alter the resulting volume and geometry of the residual limb. However, utmost care was taken in performing these morphological and smoothing operations such that the total volume of the muscle masks did not vary by more than 0.2%.

As a result of the parameters chosen for fibre tractography, and the smoothing and morphological operations that alter the geometry of the residual limb, some elements of the FE mesh might either lack fibre information or contain inconsistent fibre orientations. Incorrectly bundled fibres might also lead to inconsistent fibre directions in the mesh. The implemented fibre-correction algorithm fills in the missing fibre information and also corrects the inconsistent fibre orientations within each muscle. This however, is done at the cost of smoothing the fibre orientation field over the entire muscle domain, which might lead to over-smoothing. The extent of any such over-smoothing needs to be verified by comparing the smoothed fibre field with that of the non-smoothed, original fibre field. The error associated with manual bundling of muscle fibres also affects the fibre orientation in the muscles. Incorrectly bundling fibres of neighbouring muscles might lead to non-realistic fibre orientation at the muscle boundaries. A potential scope for improving the fibre-correction algorithm is to include checks for mutual exclusion of muscle fibre bundles. Furthermore, the Gaussian kernel size *p*, which is assumed to be constant here, can be modelled as a function of mesh density. The value of *p* should be inversely proportional to the mesh density, i.e., a dense mesh will have a smaller value of *p* than a coarse mesh. For the proposed model, these errors are expected to play a very minor role since the majority of muscle fibres in the model tend to have distinct lines of action, where relatively negligible inconsistencies and error in fibre orientations do not contribute to large differences in the direction and magnitude of the generated muscle force.

Another source of error in modelling the residual limb is the fact that tendinous structures cannot be extracted directly from DT-MRI images. But the tendinous structures are responsible for transferring the forces generated by skeletal muscles to the skeleton. However, due to the inherent limitations of the DT-MRI scans, tendons cannot be tracked. To do so, scanners with large magnetic field strength, and extremely high pixel resolution are necessary (cf. Gupta et al., 2010; Karampinos et al., 2012). In Gupta et al. (2010), excised rabbit tendons were tracked using DT-MRI with in-plane pixel resolution of 200 μm, in a 11.74 T scanner. Scanning with such high resolution is not feasible for humans due to the extremely high scan duration. As a result, models created with the proposed workflow lack tendons, which is not a limitation of the proposed methodology but rather of the current scanner technology. Tendons may, however, be laboriously hand-segmented from T1- or T2-weighted MRI scans, and imported into the limb model.

Despite these drawbacks, having good knowledge in anatomy for grouping the muscle fibres is helpful in resolving most fibre-bundling-related problems in MedINRIA. It remains to be verified in future experiments, if higher resolution DT-MRI scans will mitigate partial volume effects to such an extent that the boundaries between muscle fibres are distinctly visible, leading to easier classification of mutually exclusive fibre bundles. If not, an alternative would be to overlay the muscle masks over T1- or T2-weighted MRI scans, and correct the mask boundaries before creating the model. It is common for surgical amputations to result in extensive modifications of the limb musculature, which results in a unique residual limb anatomy in each patient. In such cases, conventional segmentation of the residual limb with MRI or CT scans, with the intention of creating a detailed limb model, is rather difficult. With the proposed technique, the anatomy of this new musculature is evident from its fibre distribution with which individual muscles of the residual limb can be easily modelled. This ease of use of the proposed workflow is obvious given the relatively short time (about 20 min) that is required to model the highly detailed patient-specific FE mesh of the stump.

### 4.2. FE mesh and continuum-mechanical model

The use of realistic constitutive laws is, like for almost any other subject-specific computational model, a challenge and a source of error. Here, the soft tissue parameters of the continuum-mechanical model were adapted from Röhrle et al. (2017), where the skeletal muscle parameters were optimised for healthy upper limb muscles. Unlike healthy muscles, amputated muscles are incapable of generating normal muscular force owing primarily to reduced physiological cross-sectional area and altered insertion points of transected muscles, among other factors. Therefore, the soft tissue parameters of the stump were adapted from the healthy parameter set by simply weakening them. The correctness of such a set is not guaranteed here. Extensive experimental methods, which currently are active research topics (cf. Sengeh et al., 2016), are required to accurately determine the correct parameter set for the stump.

Further, the contact between soft tissues in the residual limb were idealised through common nodes at the interfaces between individual parts of the FE mesh. This assumption is comparable to the role of connective tissues that bind the soft tissues together, which leads to favourable conditions in the FE analyses. For example, through the use of common nodes, frictional or sliding contact between the individual parts, which is difficult to quantify, is eliminated. This results in a quicker computation. Another benefit of this contact-less model is that there are no nonsensical gaps in-between the soft tissues and also between the bone and surrounding soft tissues during the simulations. Similarly, the sticky contact between the inner surface of the prosthetic liner worn by the subject and the skin was idealised through common nodes at their surface.

### 4.3. Boundary conditions and results of bipedal stance

The models of residual limb and prosthetic socket were generated from DT-MRI and T1-MRI scans, respectively. This was due to the fact that the socket, which lacked water content, was transparent in the DT-MRI scans and therefore could not be segmented. The correct alignment between the residual limb and socket was achieved by manually segmenting and aligning the femur in both models. This manual segmentation of the femur in Simpleware ScanIP is trivial and quick due to large differences in the pixel intensities of the bone and the surrounding soft tissues. Following this alignment procedure, the initial position of the socket for the socket donning simulation was chosen such that the stump and the socket were not in initial contact. The duration of this simulation was assumed to be 10 s, which was not experimentally-motivated. The dynamic socket donning simulation pre-conditioned the limb for the bipedal stance simulation that followed. The duration for bipedal stance was chosen to be 2 h, which is motivated by the strain-based cell necrosis studies by Gefen et al. (2008), where tolerance of tissues to compressive strains decreased significantly between 1 and 3 h upon loading.

The fused muscles, which were generated for the sake of comparison with the individual-muscle model, were created by fusing the individual muscle masks that were created with the proposed workflow. This resulted in a bone-fat-muscle complex, which still contained an equivalent or more segmentation layers than the state-of-the-art fused-muscle models, where patient-specific stump has been either segmented into bone-muscle (cf. Portnoy et al., 2008; Cagle et al., 2018) or bone-muscle-skin layers (cf. Sengeh et al., 2016). However, in the above studies, no distinction was made between fat and muscles in the soft tissue complex despite their properties being largely dissimilar. And, in order to remain consistent with the above state-of-the-art models, tissue anisotropy was ignored in the fused-muscle model.

The differences between the individual- and fused-muscle models are immediately clear upon comparing the magnitude and distribution of stresses on the muscles. A 4-fold difference in the peak stresses is present between two models, with the peak stresses in the individual- and fused-muscle models being 63.5 and 14.34 kPa, respectively. This large difference may be attributed to the contribution of passive fibre stiffness to the global stiffness matrix in the individual-muscle model, which is absent in the fused-muscle model. With regard to the stress distribution, the stresses in the fused-muscle model are easily spread over the single muscle volume, resulting in lower magnitude and larger spread of the stresses while those in the individual-muscle model are concentrated at the soft tissue interfaces. In contrary to the large stress differences in the deep tissues of the residual limb, the difference in the interface stresses between the fused- and individual-muscle models resulted in only a small difference, i.e., 5.4 kPa in the fused-muscle model and 4.9 kPa in the individual-muscle model. The magnitude of the mean interface stresses in both these models are similar to those obtained by Lacroix and Ramírez Patiño (2011). However, there is a marked difference between the peak interface and deep-tissue stresses within each model, e.g., in the individual-muscle model, the peak interface and deep-tissue stresses after the bipedal stance analysis were 24.1 and 63.5 kPa, respectively. Hence, the study of interface stresses, which is necessary to ensure good health of the skin, may not be a sufficient measure of the health of deep tissues in the stump. In the above results, the magnitude and distribution of stress in the soft tissues corresponded to those of strains, and therefore, only stresses were reported here in order to be consistent with the rest of the literature. Since the DTI model was strain-based, which in this case corresponded to the stresses, the regions of skeletal muscle predicted to be affected by DTI was comparable to the stress distribution for all three cases. As expected, bipedal stance simulation of the residual limb model with original socket predicted negligible tissue damage. However, the volume of tissues in the fused-muscle model that was predicted to have been damaged was less than half of that predicted to be damaged in the individual-muscle model. The proposed hypothesis that individual-muscle models are advantageous and provide more insight in stump-prosthesis interaction studies is hereby verified.

## 5. Conclusion

The focus of this work was on developing an efficient modelling-simulation-analysis workflow to investigate stump-socket interaction during bipedal stance using patient-specific, three-dimensional, continuum-mechanical, finite element residual limb model, in which the model is generated with minimal user intervention. A nonlinear, hyperelastic, transversely isotropic continuum-mechanical model of the soft tissues was implemented in LS-DYNA to measure stresses in the residual limb. With the implemented FE model, potential sites of deep tissue injury were predicted. Comparison of the proposed individual-muscle model with the state-of-the-art residual fused-muscle/fat models reveals that the fused-muscle models underestimate the stresses and volume of injured tissues. Tissue anisotropy contributes to both active and passive stresses, and must be considered when studying socket-stump interactions. Moreover, for forward dynamics simulations, the proposed model provides an efficient method to obtain FE models of skeletal muscles, including its fibre orientations. In the forthcoming studies, it is envisioned to extend this workflow to model tendons and to include the effects of muscle contractions for various dynamic boundary conditions.

## Ethics statement

This study was carried out in accordance with the recommendations of ICH-GCP guidelines of, The ethics committee of the medical faculty of University of Tübingen and University Hospital Tübingen with written informed consent from all subjects. All subjects gave written informed consent in accordance with the Declaration of Helsinki. The protocol was approved by the the ethics committee of the medical faculty of University of Tübingen and University Hospital Tübingen.

## Author contributions

ER worked out most of the technical details, performed the numerical simulations and its analysis, and drafted the manuscript. ER and BD performed the image processing while OA was involved in implementing the constitutive laws. S-YC and LG were in charge of the data collection, and GS and FS were in charge of the MRI imaging. OR devised, conceptualised and supervised the project and drafted parts of the manuscript. All authors provided critical feedback, and agree with the content of the manuscript.

### Conflict of interest statement

The authors declare that the research was conducted in the absence of any commercial or financial relationships that could be construed as a potential conflict of interest. The reviewer AD declared a past collaboration with two of the authors, ER and OR, to the handling Editor.
